# GEPSI: A Gene Expression Profile Similarity-Based Identification Method of Bioactive Components in Traditional Chinese Medicine Formula

**DOI:** 10.1155/2018/6935350

**Published:** 2018-03-06

**Authors:** Baixia Zhang, Shuaibing He, Chenyang Lv, Yanling Zhang, Yun Wang

**Affiliations:** ^1^College of Chinese Medicine, Hebei University, Hebei, Baoding 071002, China; ^2^School of Chinese Materia Medica, Beijing University of Chinese Medicine, Beijing 100102, China; ^3^Chinese Academy of Medical Sciences and Peking Union Medical College Institute of Medicinal Plant Development, Beijing 100193, China

## Abstract

The identification of bioactive components in traditional Chinese medicine (TCM) is an important part of the TCM material foundation research. Recently, molecular docking technology has been extensively used for the identification of TCM bioactive components. However, target proteins that are used in molecular docking may not be the actual TCM target. For this reason, the bioactive components would likely be omitted or incorrect. To address this problem, this study proposed the GEPSI method that identified the target proteins of TCM based on the similarity of gene expression profiles. The similarity of the gene expression profiles affected by TCM and small molecular drugs was calculated. The pharmacological action of TCM may be similar to that of small molecule drugs that have a high similarity score. Indeed, the target proteins of the small molecule drugs could be considered TCM targets. Thus, we identified the bioactive components of a TCM by molecular docking and verified the reliability of this method by a literature investigation. Using the target proteins that TCM actually affected as targets, the identification of the bioactive components was more accurate. This study provides a fast and effective method for the identification of TCM bioactive components.

## 1. Introduction

A method to identify the bioactive components in traditional Chinese medicine (TCM) from their complex mixtures is a critical challenge of TCM research. Because of its intuitive and efficient characteristics, molecular docking has become an important means for the identification of TCM bioactive components. The basis of identification via molecular docking involves one or multiple target proteins and the components being screened; ultimately, the components that specifically act on target protein can be identified, such as TCM bioactive components. In the screening process, a single target or multiple targets are chosen, usually targets associated with a specific disease. Methods for choosing targets are generally based on a database of disease-associated targets, a key target in a signaling transduction network or from the literature [1–3]. Because of the complexity of a disease, multiple targets may be associated with it. Therefore, the target proteins selected may not be the actual targets affected by TCM, or it may not be possible to screen against all of the associated targets. Therefore, the bioactive components obtained by molecular docking may not be the components that actually cured the corresponding disease or have been left out.

The development of chemical informatics and bioinformatics has led to the accumulation of data on TCM components, target proteins, and gene expression profiles. To determine a method for the selection of target proteins for molecular docking guided by the ideas of system pharmacology, this study proposed a method for determining the target proteins of TCM and then identified the bioactive components of TCM by molecular docking. This method has been designated the gene expression profile similarity-based identification (GEPSI) method. The basic concept is to choose the gene expression profiles that are targeted by small molecule drugs in Cmap based on the principle that they have higher comparability with the gene expression profiles of a TCM, and calculate the gene expression profiles similarity between the TCM and the small molecule drugs. The target proteins of the small molecule drugs that have higher similarity scores could be considered TCM targets. Aiming at these target proteins, virtual screening is carried out to screen the TCM components, ultimately identifying the bioactive components. Because it considers the entirety of the TCM components and all of the genes affected as the object, this method could embody the holistic thinking of TCM research more concretely. This method provides an effective means for the identification of TCM bioactive components and could serve as a basis for drug repositioning, quality control, and TCM drug design.

## 2. Methods and Materials

### 2.1. Principle of GEPSI

**Figure 1:**
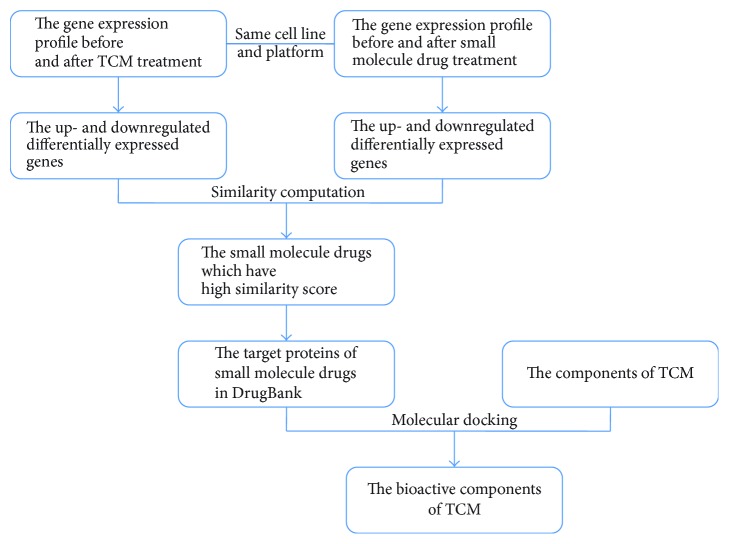
The workflow of GEPSI.

Both TCMs and small molecule drugs all can affect gene expression. By comparing the gene expression profiles before and after treatment with TCM or a small molecule drug, up- and downregulated differentially expressed genes can be identified. Then, these up and downregulated differentially expressed genes that are affected by TCMs and small molecule drugs can be compared, and a similarity score can be obtained. If the similarity score is high, the TCM and the small molecule drug may have similar pharmacological action, and the target proteins for the small molecule drugs that have higher similarity score can be considered targets for the TCM. Using these proteins as the targets, we can finally identify the bioactive components of a TCM by molecular docking. We also discuss each step of the ITPI method (Figure 1) in detail in this paper.

#### 2.1.1. Gene Expression Profile Data

The Gene Expression Omnibus (GEO, https://www.ncbi.nlm.nih.gov/geo/) contains some gene expression profiles which were treated with components, herbs, and TCM formulae. These data were utilized to carry out TCM-related research. Connectivity Map (CMap, https://www.broadinstitute.org/cmap/) is a gene expression profile database related to small molecule drugs [34]. Cmap establishes the relations of small molecule drugs, genes, and diseases according to the gene expression differences in human tumor cell lines after treatment with small molecule drugs. By comparing the similarity of different gene expression profiles, Cmap is mainly applicable to the areas of drug development, such as drug repositioning. Different human tumor cell lines (HL60, MCF7, PC3, SKMEL5, and ssMCF7) were treated with small molecules drugs at different concentrations (10 nM, 100 nM, 1 *μ*M, and 10 *μ*M) for different times (6 h, 12 h). At present, Cmap contains data for 1309 small molecule drugs and more than 7000 gene expression profiles. Of these 1309 drugs, 556 drugs were recorded in DrugBank. Of these 556 drugs, 522 drugs had the data of target protein. This study chose gene expression profiles that had high similarity to TCMs with respect to the same cell lines types and platforms.

#### 2.1.2. The Determination of Up and Downregulated Genes

The differentially expressed genes were determined using the bioinformatics toolbox of Matlab [35]. A* t*-test and false discovery rate (FDR) of multiple hypothesis testing were performed on each gene. Significant differentially expressed genes were detected by random sample replacement (*P* < 0.05, FDR ≤ 0.1). Up and downregulated genes were distinguished by the magnitude of fold change (FC). If FC ≥ 2, then the significant differentially expressed genes were up-regulated genes, and if FC ≤ 0.5, then the significant differentially expressed genes were downregulated genes.

#### 2.1.3. The Similarity Computation of the Gene Expression Profile

The up- and downregulated genes were used to calculate the gene expression profile similarity. Using up and downregulated genes as the base data, the gene expression profile similarity was automatically calculated in Cmap by the K-S algorithm [34, 36]. A similarity comparison yielded the similarity scores of the gene expression profiles of each small molecule drugs and TCM. Similarity scores fell between −1 and 1. If 0 ≤ similarity scores ≤ 1, the pharmacological action of a small molecule drug and TCM were similar, and a higher absolute value of the similarity score indicated a greater similarity; if −1 ≤ similarity scores ≤ 0, the pharmacological action of a small molecule drug and TCM were adverse, and a higher absolute value indicated less similarity.

#### 2.1.4. Determination of the TCM Target Proteins

If the similarity score for the gene expression profiles of a small molecule drug and TCM was high, then their pharmacological action was similar. The target proteins of a small molecule drugs were considered TCM targets. This study only considered the top 10 small molecule drugs that had a definite pharmacological action and their target proteins were recorded in DrugBank version 4.3.

#### 2.1.5. Data for the TCM Components

The components of a TCM formula were collected from TCMD [37] and TCMSP [38]. The components were supplemented and perfected by the literature in CNKI and PubMed (1979~2017). The name, structure, and SMILES string of a component was recorded. For components with synonyms, the repetitive components were deleted by the “full structure” algorithm in “ChemBioFinder for Office 12.0”.

#### 2.1.6. Determination of Bioactive Components of a TCM

The three-dimensional structure was downloaded from the PDB (https://www.rcsb.org/pdb/home/home.do), and the structure that had active ligands and higher resolution was preferentially selected. The preprocessing of the target protein included the deletion of ligands, water, and redundant protein conformations; the completion of missing or incomplete residues; the addition of hydrogens; and the distribution of related charges. The amino acids in the target protein that interact with the ligand were selected and were defined as the active pocket. The structure of components was transformed into a three-dimensional structure, endowed with a CHARMM force field and protonated in accordance with the corresponding pH. Molecular docking was carried out by LibDock [39], and the parameter settings were as follows: the “Conformation Method” was “BEST,” the “Docking Preferences” was “High Quality,” and the other parameters were set to the default. With the “LibDock Score” as the reference, the components that had a score higher than the ligand and the ranked in the top 10 were considered the bioactive components. This information allowed us to identify the bioactive components of the TCM.

### 2.2. The Application of GEPSI on SWT

Si-Wu-Tang (SWT) is a well-known TCM formula and is prepared from four medicinal herbs including* Rehmanniae Radix Praeparata* (*Rehmannia glutinosa* Libosch.),* Angelicae Sinensis Radix* (*Angelica sinensis* (Oliv.) Diels),* Paeoniae Radix Alba* (*Paeonia lactiflora* Pall.), and* Chuanxiong Rhizoma* (*Ligusticum chuanxiong* Hort.).* SWT* and its series of decoctions (i.e., the Xiang-Fu-Si-Wu decoction, and the Tao-Hong-Si-Wu decoction) have been widely used in clinical gynecology practice for blood stasis syndrome, such as primary dysmenorrheal, breast cancer, and other estrogen-related diseases [40–44]. For* SWT*, this study applied the TCM bioactive components identification method based on the similarity of the gene expression profiles. In GEO, the number of gene expression profiles that* SWT* (0.0256 mg/mL, 0.256 mg/mL, and 2.56 mg/mL) acted on, MCF-7, was GSE23610. GSE23610 was obtained on the GPL570 platform (HG-U133_Plus_2) [45]. A total of 3905 gene expression profiles were selected in Cmap for the same cell line (MCF-7) and platform (HG-U133_Plus_2). These gene expression profiles involved 1294 small molecule drugs. In addition, 98 components of* Rehmanniae Radix Praeparata*, 215 components of* Angelicae Sinensis Radix*, 85 components of* Paeoniae Radix Alba*, and 258 components of* Chuanxiong Rhizoma* were collected. The collected components can be seen in the Supplemental Information 1.

## 3. Results and Discussions

### 3.1. Up- and Downregulated Genes

At* SWT* concentrations of 0.0256 mg/mL and 0.256 mg/mL, the expression of each gene did not obviously change, but when the* SWT* concentration was 2.56 mg/mL, the expression of each gene obviously changed. Therefore, the gene expression profile that was elicited by* SWT *(2.56 mg/mL) was chosen for follow-up research.

A* t*-test and false discovery rate (FDR) multiple hypothesis test were applied to each gene. A large number of genes were found to have biological differences; 442 genes were up-regulated and 189 were downregulated (Supplemental Information 2).

### 3.2. The Small Molecule Drugs with High Similarity Scores

After the similarity was computed, the similarity scores of the gene expression profiles for 1294 small molecular drugs and* SWT* were obtained. The top ten small molecule drugs that had explicit pharmacological action and their target proteins contained in DrugBank were retained. The results are shown in Table 1.

### 3.3. The Primary Pharmacological Actions of the Top Ten Small Molecule Drugs

The primary pharmacological actions of the top ten small molecule drugs in Table 1 were investigated in the literature. The results are shown in Table 2.

Table 2 shows that the pharmacological actions of the ten small molecule drugs all involve disease caused by an unbalanced estrogen level. Except for phenoxybenzamine and equilin, the primary pharmacological action of the remaining eight small molecule drugs was closely related to the treatment of breast cancer. Most of the eight drugs have an estrogenic effect. For example, resveratrol and genistein are phytoestrogens; estradiol is a natural estrogen that is secreted by mature ovarian follicles; diethylstilbestrol is a kind of estrogen that is a common endocrine medication for breast cancer. We often think that the occurrence of breast cancer is related to an excessive or imbalanced level of estrogen in the female body [46], and the regulation of immunity is an important method for the treatment of cancer. To summarize,* SWT* may have an anti-breast cancer effect because it has a high similarity score with the top ten small molecule drugs.

### 3.4. The Target Proteins That Were Used in Molecular Docking

Of the top ten small molecule drugs, only the target proteins of four drugs have a three-dimensional structure in the PDB with a high resolution and corresponding bioactive ligands. Therefore, the target proteins of these four drugs were used for molecular docking studies (Table 3).

### 3.5. Bioactive Components of SWT

After molecular docking, the components whose LibDock score were higher than that of the ligand were identified as bioactive components (the LibDock score of the ligand is shown in Supplemental Information 3). This study identified 46 bioactive components, including 12 components in* Paeoniae Radix Alba*, 4 components in* Chuanxiong Rhizoma*, 6 components in* Angelicae Sinensis Radix,* and 24 components in* Rehmanniae Radix Praeparata* (the results are shown in Table 4). The 46 bioactive components act on 9 target proteins.

### 3.6. Verification of the Reliability of GEPSI

Table 4 shows that* SWT* has anti-breast cancer activity through 46 bioactive components and these components acted on 9 target proteins. Most of the bioactive components, such as catalpol, verbascoside, and paeoniflorin, acted on multiple targets. The types and numbers of targets that the bioactive components acted on were diverse. If we only use one or a few proteins as targets, the bioactive components retrieved may be not complete. For example, 19 bioactive components, such as catalpol, aucubin, and melittoside will be not be retrieved when ISGO is the target protein. Therefore, we should consider all the targets that TCM could affect when a comprehensive bioactive components screening is carried out.

The proteins in Table 4 were the targets of resveratrol, diethylstilbestrol, estradiol, and genistein. According to the literature, these four components were estrogen or had an estrogen-like effect, and resveratrol and genistein had potent anti-breast cancer activity. Hence, the 46 bioactive components of* SWT* may also have anti-breast cancer and estrogen-like effects. Studies showed that catalpol, a DNA polymerase inhibitor, inhibited the proliferation of six human solid tumor cell lines by acting during the G0-G1 period. The naturally occurring iridoid catalpol is a Taq DNA polymerase inhibitor. However, the formation of analogs bearing one to three silyl ether groups led to antiproliferative compounds against a panel of six human solid tumor cell lines, with GI50 values in the range 1.8–4.8 *μ*M. Cell cycle studies revealed an arrest of the G0/G1 phase that was consistent with DNA polymerase inhibition [47]. Orientin could suppress the proliferation of MCF-7 and present specific dose-response relationships [48, 49]. Paeoniflorin could suppress the proliferation and spread of breast cancer cells through the Notch-1 pathway [50]. The effect of trigalacturonic acid on the proliferation inhibition of Bcap-3 in breast cancer cells was better and it may have an anti-breast cancer potential [51]. To summarize, we found some bioactive components did have the same effect as small molecule drugs via a literature research which indicated the reliability of the bioactive components identification method based on the similarity of gene expression profiles.

## 4. Conclusion

This method was more accurate with the protein that TCM actually acted on as the target, and the result was more comprehensive than a determination of the target protein according to disease-related target databases and signal transduction networks. For example, there are 74 breast cancer-related targets in the Therapeutic Target Database (TTD), including the estrogen receptor (ER), the vascular endothelial growth factor receptor 1 (VEGFR1), and the epidermal growth factor receptor (EGFR). However, no evidence was available to support the selection of these proteins as targets. This study identified the target protein that* SWT* actually acted on by a gene expression similarity comparison, identified all the bioactive components of* SWT* by molecular docking, and then verified the reliability of this method through a literature investigation. GEPSI could serve as a rapid and effective method for the identification of TCM bioactive components. Although some time is necessary to perfect related databases, such as components of TCM and the target protein structure of small drugs, we believe the data that used in GEPSI will be more complete, and the results will be more accurate with the development of chemical informatics and bioinformatics.

Meanwhile, this study has also revealed that* SWT* had anti-breast cancer efficacy. However, there have been no studies of these effects. The Tao-hong Si-wu Decoction, a derivative formula, was proved to influence the upper limb swelling after breast cancer surgery and the quality of a chemotherapy patient's life [52, 53]. Research has shown that* Paeoniae Radix Alba*,* Chuanxiong Rhizoma*,* Rehmanniae Radix Praeparata, *and* SWT* have plant estrogen-like effects, but the bioactive components have not been identified [54]. The above studies indirectly illustrate the rationality that* SWT* has an anti-breast cancer effect. That is to say, GEPSI also can be used for drug repositioning. Now that the bioactive components have been identified, we can control the quality of the individual herbs. We can also design an anti-breast cancer drug combination based on the bioactive components in* SWT*.

## Figures and Tables

**Table 1:** Top ten small molecule drugs that have higher similarity scores with the gene expression profile of *SWT*.

Cmap name	Mean	Enrichment	*P* value	Percent nonnull	Group in DrugBank
Phenoxybenzamine	0.955	1.000	0.00000	100	Approved
Diethylstilbestrol	0.703	0.983	0.00004	100	Approved
Anisomycin	0.695	0.990	0.00012	100	Experimental
Equilin	0.609	0.970	0.00004	100	Approved
Digoxigenin	0.609	0.950	0.00012	100	Approved
Resveratrol	0.413	0.855	0.00002	100	Experimental/investigational
Estradiol	0.389	0.774	0.00000	94	Approved/investigational
Prochlorperazine	0.376	0.674	0.00012	88	Approved
Genistein	0.352	0.686	0.00000	81	Investigational
Thioridazine	0.344	0.626	0.00004	72	Approved

**Table 2:** The primary pharmacological action of the top ten small molecule drugs.

Drug	Primary pharmacological action	References
Phenoxybenzamine	Hypertension; mediated peripheral vasodilation; pheochromocytoma	[4–6]
Diethylstilbestrol	Menopausal syndrome; postmenopausal osteoporosis; breast cancer	[7–9]
Anisomycin	Immunosuppression	[10, 11]
Equilin	Postmenopausal osteoporosis	[12]
Digoxigenin	Hypertension; valvular heart disease; cell proliferation inhibition, such as breast cancer, etc.	[13–16]
Resveratrol	Anticancer; immunoregulation	[17–20]
Estradiol	Metastatic breast cancer	[21–23]
Prochlorperazine	Antinausea after chemotherapy	[24, 25]
Genistein	Anticancer; radiation protection; immunoregulation	[26–29]
Thioridazine	Anticancer; antischizophrenia	[30–33]

**Table 3:** The target proteins of small molecule drugs that were used for molecular docking studies.

Drug	Mode of action	The Uniprot number	Abbreviation of protein	The PDB number
Diethylstilbestrol	Agonist	Q92731	ESR2	1QKM
	Agonist	P03372	ESR1	1X7R
	Unknown	O75469	NR1I2	4X1F

Resveratrol	Unknown	P03372	ESR1	1QKT
	Unknown	Q92731	ESR2	4J24

Estradiol	Agonist	P16083	NQO2	1SG0
	Agonist	P68400	CSNK2A1	4RLL

Genistein	Unknown	P03372	ESR1	3ERD
	Unknown	P62508	ESRRG	2GPP

**Table 4:** The 46 bioactive components of *SWT*.

Herb	Compound	PubChem CID	LibDock score	Target protein
Shudi	Catalpol	91520	130.006	1QKM
	Catalpol	91520	127.034	1X7R
	Catalpol	91520	119.571	3ERD
	Catalpol	91520	120.630	4J24
	Isoacteoside	6476333	176.387	4RLL
	Isoacteoside	6476333	136.387	ISGO
	Leucosceptoside A	10394343	134.596	ISGO
	aucubin	91458	119.278	3ERD
	Cistanoside F	44429870	143.468	1QKT
	Cistanoside F	44429870	127.692	ISGO
	Melittoside	11968737	151.290	4X1F
	Acteoside	5281800	178.203	4RLL
	Acteoside	5281800	150.245	ISGO
	Martynoside	44429856	148.239	ISGO
	Forsythoside A	45358127	178.534	4RLL
	Forsythoside A	45358127	146.041	ISGO
	Ajugoside	179611	119.032	4J24
	Jioglutoside B	11968648	140.384	4X1F
	Jioglutoside B	11968648	123.671	ISGO
	Jioglutoside A	11968647	116.065	3ERD
	Jionoside D	9895632	130.081	ISGO
	Echinacoside	5281771	134.559	ISGO
	Dihydrocatalpol	5705531	128.987	1QKM
	Glutinoside	24884124	131.718	1X7R
	Glutinoside	24884124	122.022	3ERD
	Rehmannioside C	6325883	149.106	1QKT
	Rehmannioside C	6325883	137.092	4J24
	Rehmannioside C	6325883	153.335	4X1F
	Rehmannioside C	6325883	135.653	ISGO
	Rehmannioside B	6325882	145.167	1QKT
	Rehmannioside B	6325882	143.713	4X1F
	Rehmannioside B	6325882	126.837	ISGO
	Rehmannioside A	86287413	142.582	1QKT
	Rehmannioside A	86287413	138.007	4X1F
	Rehmannioside A	86287413	138.555	ISGO
	Daucosterol	5742590	138.157	4X1F
	Daucosterol	5742590	135.135	ISGO
	Geniposide	107848	129.014	1X7R
	Forsythiaside	5281773	177.534	4RLL
	Forsythiaside	5281773	148.451	ISGO
	8-Epiloganic acid	158144	128.867	1X7R
	8-Epiloganic acid	158144	121.873	3ERD
	sec-Hydroxyaeginetic acid	15693867	129.657	1QKM
	sec-Hydroxyaeginetic acid	15693867	139.930	1QKT
	sec-Hydroxyaeginetic acid	15693867	119.597	3ERD
	sec-Hydroxyaeginetic acid	15693867	119.688	4J24
	sec-Hydroxyaeginetic acid	15693867	177.998	4RLL
	sec-Hydroxyaeginetic acid	15693867	149.140	4X1F
	sec-Hydroxyaeginetic acid	15693867	149.648	ISGO

Danggui	Orientin	5281675	126.131	4J24
	Orientin	5281675	128.047	ISGO
	Trigalacturonic acid	3641243	141.911	4X1F
	Sphingomyelin	52931203	121.153	3ERD
	Daucosterol	5742590	138.157	4X1F
	Daucosterol	5742590	135.135	ISGO
	Senkyunolide	91731751	170.306	4RLL
	1,1,5-Trimethyl-2-formyl- cyclohea-2,5-diene-4-one	None	140.619	1QKT
	1,1,5-Trimethyl-2-formyl- cyclohexa-2,5-diene-4-one	None	142.328	2GPP
	1,1,5-Trimethyl-2-formyl- cyclohexa-2,5-diene-4-one	None	123.128	4J24
	1,1,5-Trimethyl-2-formyl- cyclohexa-2,5-diene-4-one	None	126.443	ISGO
	1,1,5-Trimethyl-2-formyl- cyclohexa-2,5-diene-4-one	None	125.665	ISGO

Chuanxiong	Heptadecanoic acid	10429233	127.735	1X7R
	Heptadecanoic acid	10429233	124.190	4J24
	Butyraldehyde	14900	122.060	3ERD
	Daucosterol	5742590	138.157	4X1F
	Daucosterol	5742590	135.135	ISGO
	Methyl 3,4-dimethylbenzoate	7852	140.619	1QKT
	Methyl 3,4-dimethylbenzoate	7852	123.128	4J24
	Methyl 3,4-dimethylbenzoate	7852	125.665	ISGO

Baishao	Paeonianin E	44256843	141.990	ISGO
	Peonin	44253993	135.516	4X1F
	Peonin	44253993	124.317	ISGO
	Stigmasterol Glucoside	6440962	134.691	ISGO
	Paeonoside	52952637	172.449	4RLL
	Paeonoside	52952637	133.551	ISGO
	Paeoniflorin	442534	149.657	1QKT
	Paeoniflorin	442534	126.988	1X7R
	Paeoniflorin	442534	122.872	4J24
	Paeoniflorin	442534	138.060	4X1F
	Oxypaeoniflorin	429559	146.127	1QKT
	Oxypaeoniflorin	429559	141.528	4X1F
	Methyl linolelaidate	5362793	116.328	3ERD
	Galloylpaeoniflorin	46882879	137.131	ISGO
	Dotriacontane	11008	127.379	ISGO
	Acetytastragaloside	5282102	129.735	ISGO
	Albiflorin	162355	143.965	1QKT
	1,2,3,6-Tetragalloylglucose	73178	137.874	ISGO

## Supplementary Materials

Supplementary Materials Supplementary Materials 1: it includes 656 components of SWT. These components were used for molecular docking. Supplementary Materials 2: 442 upregulated genes and 189 downregulated genes. Supplementary Materials 3: the LibDock score of the ligand.

## References

[1] Wang Y., Hu J.-S., Lin H.-Q., Ip T.-M., Wan D. C.-C. (2016). Herbalog: A tool for target-based identification of herbal drug efficacy through molecular docking. *Phytomedicine*.

[2] Feng L. X., Jing C. J., Tang K. L. (2011). Clarifying the signal network of salvianolic acid B using proteomic assay and bioinformatic analysis. *Proteomics*.

[3] Chen L., Du J., Dai Q., Zhang H., Pang W., Hu J. (2014). Prediction of anti-tumor chemical probes of a traditional Chinese medicine formula by HPLC fingerprinting combined with molecular docking. *European Journal of Medicinal Chemistry*.

[4] Agrawal R., Mishra S. K., Bhatia E. (2014). Prospective study to compare peri-operative hemodynamic alterations following preparation for pheochromocytoma surgery by phenoxybenzamine or prazosin. *World Journal of Surgery*.

[5] Te A. E. (2002). A modern rationale for the use of phenoxybenzamine in urinary tract disorders and other conditions. *Clinical Therapeutics*.

[6] Ganguly A., Weinberger M. H., Fineberg N. S. (1983). Cardiovascular, humoral, and renal effects of phenoxybenzamine in hypertension. *American Journal of Kidney Diseases*.

[7] Chen H., Geng C. Z., Kuang G. (2007). Diethylstilbestrol intervention carcinogenesis of breast cancer in wistar rats. *Chinese journal of cancer*.

[8] Lin S. E., Huang J. P., Wu L. Z., Wu T., Cui L. (2013). Prevention of osteopenia and dyslipidemia in rats after ovariectomy with combined aspirin and low-dose diethylstilbestrol. *Biomedical and Environmental Sciences*.

[9] Geisler J., Haynes B., Anker G. (2005). Treatment with high-dose estrogen (diethylstilbestrol) significantly decreases plasma estrogen and androgen levels but does not influence in vivo aromatization in postmenopausal breast cancer patients. *The Journal of Steroid Biochemistry and Molecular Biology*.

[10] Monaghan D., O'Connell E., Cruickshank F. L. (2014). Inhibition of protein synthesis and JNK activation are not required for cell death induced by anisomycin and anisomycin analogues. *Biochemical and Biophysical Research Communications*.

[11] Schmeits P. C. J., Katika M. R., Peijnenburg A. A. C. M., van Loveren H., Hendriksen P. J. M. (2014). DON shares a similar mode of action as the ribotoxic stress inducer anisomycin while TBTO shares ER stress patterns with the ER stress inducer thapsigargin based on comparative gene expression profiling in Jurkat T cells. *Toxicology Letters*.

[12] Lobo R. A., Nguyen H. N., Eggena P., Brenner P. F. (1988). Biologic effects of equilin sulfate in postmenopausal women. *Fertility and Sterility*.

[13] Zhang H. F., Qian D. Z., Tan Y. S. (2008). Digoxin and other cardiac glycosides inhibit HIF-1a synthesis and block tumor growth. *PNAS*.

[14] Samanta D., Gilkesa D. M., Chaturvedia P., Xiang L., Semenza G. L. (2014). Hypoxia-inducible factors are required for chemotherapy resistance of breast cancer stem cells. *Proceedings of the National Acadamy of Sciences of the United States of America*.

[15] Damoiseaux J. G. M. C., Theunissen R., Broeren C. P. M., Van Breda Vriesman P. J. C., Duijvestijn A. M. (1998). Comparison of detection techniques for cytokine reverse transcriptase polymerase chain reaction; Digoxigenin-labeled polymerase chain reaction permits sensitive detection of cytokine mRNA in rat heart allografts. *Journal of Immunological Methods*.

[16] Akera T., Wiest S. A., Brody T. M. (1979). Differential effect of potassium on the action of digoxin and digoxigenin in guinea-pig heart. *European Journal of Pharmacology*.

[17] Khan A., Aljarbou A. N., Aldebasi Y. H., Faisal S. M., Khan M. A. (2014). Resveratrol suppresses the proliferation of breast cancer cells by inhibiting fatty acid synthase signaling pathway. *Cancer Epidemiology*.

[18] Kim T. H., Shin Y. J., Won A. J. (2014). Resveratrol enhances chemosensitivity of doxorubicin in multidrug-resistant human breast cancer cells via increased cellular influx of doxorubicin. *Biochimica et Biophysica Acta (BBA) - General Subjects*.

[19] Gomez L. S., Zancan P., Marcondes M. C. (2013). Resveratrol decreases breast cancer cell viability and glucose metabolism by inhibiting 6-phosphofructo-1-kinase. *Biochimie*.

[20] Lai X., Pei Q., Song X. (2016). The enhancement of immune function and activation of NF-*κ*B by resveratrol-treatment in immunosuppressive mice. *International Immunopharmacology*.

[21] Chalasani P., Stopeck A., Clarke K., Livingston R. (2014). A pilot study of estradiol followed by exemestane for reversing endocrine resistance in postmenopausal women with hormone receptor-positive metastatic breast cancer. *The Oncologist*.

[22] Tao S., He H., Chen Q. (2015). Estradiol induces HOTAIR levels via GPER-mediated miR-148a inhibition in breast cancer. *Journal of Translational Medicine*.

[23] Wisinski K. B., Xu W., Tevaarwerk A. J. (2016). Targeting Estrogen Receptor Beta in a Phase 2 Study of High-Dose Estradiol in Metastatic Triple-Negative Breast Cancer: A Wisconsin Oncology Network Study. *Clinical Breast Cancer*.

[24] Silvey L., Carpenter J. T., Wheeler R. H., Lee J., Conolley C. (1988). A randomized comparison of haloperidol plus dexamethasone versus prochlorperazine plus dexamethasone in preventing nausea and vomiting in patients receiving chemotherapy for breast cancer. *Journal of Clinical Oncology*.

[25] Markman M., Sheidler V., Ettinger D. S., Quaskey S. A., Mellits E. D. (1984). Antiemetic efficacy of dexamethasone. Randomized, double-blind, crossover study with prochlorperazine in patients receiving cancer chemotherapy. *The New England Journal of Medicine*.

[26] Chen J., Duan Y., Zhang X., Ye Y., Ge B., Chen J. (2015). Genistein induces apoptosis by the inactivation of the IGF-1R/p-Akt signaling pathway in MCF-7 human breast cancer cells. *Food & Function*.

[27] Avci C. B., Susluer S. Y., Caglar H. O. (2015). Genistein-induced mir-23b expression inhibits the growth of breast cancer cells. *Wspolczesna Onkologia*.

[28] Terra V. A., Souza-Neto F. P., Frade M. A. C. (2015). Genistein prevents ultraviolet B radiation-induced nitrosative skin injury and promotes cell proliferation. *Journal of Photochemistry and Photobiology B: Biology*.

[29] Kogiso M., Sakai T., Mitsuya K., Komatsu T., Yamamoto S. (2006). Genistein suppresses antigen-specific immune responses through competition with 17*β*-estradiol for estrogen receptors in ovalbumin-immunized BALB/c mice. *Nutrition Journal *.

[30] Yin T., He S., Shen G., Ye T., Guo F., Wang Y. (2015). Dopamine receptor antagonist thioridazine inhibits tumor growth in a murine breast cancer model. *Molecular Medicine Reports*.

[31] Smith R. C., Baumgartner R., Ravichandran G. K. (1984). Plasma and red cell levels of thioridazine and clinical response in schizophrenia. *Psychiatry Research*.

[32] Strobl J. S., Kirkwood K. L., Lantz T. K., Lewine M. A., Peterson V. A., Worley J. F. (1990). Inhibition of Human Breast Cancer Cell Proliferation in Tissue Culture by the Neuroleptic Agents Pimozide and Thioridazine. *Cancer Research*.

[33] Sachlos E., Risueño R. M., Laronde S. (2012). Identification of drugs including a dopamine receptor antagonist that selectively target cancer stem cells. *Cell*.

[34] Lamb J., Crawford E. D., Peck D. (2006). The connectivity map: using gene-expression signatures to connect small molecules, genes, and disease. *Science*.

[35] Clark N. R., Hu K. S., Feldmann A. S. (2014). The characteristic direction: A geometrical approach to identify differentially expressed genes. *BMC Bioinformatics*.

[36] Ene M. T. (1999). Nonparametric Statistical Methods. *Statistics in Medicine*.

[37] Chen C. Y. (2011). TCM Database@Taiwan: the world's largest traditional Chinese medicine database for drug screening *in silico*. *PLoS ONE*.

[38] Ru J., Li P., Wang J. (2014). TCMSP: a database of systems pharmacology for drug discovery from herbal medicines. *Journal of Cheminformatics*.

[39] Sarvagalla S., Singh V. K., Ke Y.-Y. (2015). Identification of ligand efficient, fragment-like hits from an HTS library: Structure-based virtual screening and docking investigations of 2H- and 3H-pyrazolo tautomers for Aurora kinase A selectivity. *Journal of Computer-Aided Molecular Design*.

[40] Liu P., Li W., Li Z.-H. (2014). Comparisons of pharmacokinetic and tissue distribution profile of four major bioactive components after oral administration of Xiang-Fu-Si-Wu Decoction effective fraction in normal and dysmenorrheal symptom rats. *Journal of Ethnopharmacology*.

[41] Miao E. Y., Miao M. Y.-M., Kildea D. G., Lao Y.-W. (2014). Effects of electroacupuncture and electroacupuncture plus Tao Hong Si Wu Wan in treating primary dysmenorrhea. *JAMS Journal of Acupuncture and Meridian Studies*.

[42] Liu P., Duan J.-A., Hua Y.-Q., Tang Y.-P., Yao X., Su S.-L. (2011). Effects of Xiang-Fu-Si-Wu Decoction and its main components for dysmenorrhea on uterus contraction. *Journal of Ethnopharmacology*.

[43] Yue G., Zengchun M., Qiande L. (2010). Combined Research of Si wu tang and Blood Deficiency. *World Science and Technology*.

[44] Liu L., Ma H. Y., Tang Y. P. (2012). Discovery of estrogen receptor a modulators from natural compounds in Si-Wu-Tang series decoctions using estrogen-responsive MCF-7 breast cancer cells. *Bioorganic & Medicinal Chemistry Letters*.

[45] Wen Z., Wang Z., Wang S. (2011). Discovery of molecular mechanisms of traditional Chinese medicinal formula Si-Wu-Tang using gene expression microarray and connectivity map. *PLoS ONE*.

[46] Yu K.-D., Rao N.-Y., Chen A.-X., Fan L., Yang C., Shao Z.-M. (2011). A systematic review of the relationship between polymorphic sites in the estrogen receptor-beta (ESR2) gene and breast cancer risk. *Breast Cancer Research and Treatment*.

[47] Pungitore C. R., Leon L. G., Carcia C. (2007). Novel antiproliferative analogs of the Taq DNA polymerase inhibitor catalpol. *Bioorganic & Medicinal Chemistry Letters*.

[48] Jiang W. (2013). *Studies on synthesis and structure characterization and biological activity of orientin-zinc complexes of trollius chinensis bunge*.

[49] Zu Y.-G., Liu X.-L., Fu Y.-J., Wu N., Kong Y., Wink M. (2010). Chemical composition of the SFE-CO2 extracts from Cajanus cajan (L.) Huth and their antimicrobial activity in vitro and in vivo. *Phytomedicine*.

[50] Zhang Q., Yuan Y., Cui J., Xiao T., Jiang D. (2016). Paeoniflorin inhibits proliferation and invasion of breast cancer cells through suppressing Notch-signaling pathway. *Biomedicine & Pharmacotherapy*.

[51] Zhang Y. Y., Mu T. H., Zhang M. (2012). Effects of modified sweet potato pectins on the proliferation of cancer cells. *Scientia Agricultura Sinica*.

[52] Yan S. F., Wang S. X., Zhou Y. B. (2009). Treatment of 91 cases of edematous upper limb following mammary cancer surgery by, Taohong Siwu Dicoction. *Shanghai journal of traditional Chinese medicine*.

[53] Dong J. (2014). Effects of Tao-hong Si-wu Decoction Combined with Neoadjuvant Chemotherapy on. *Guiding Journal of Traditional Chinese Medicine and Pharmacy*.

[54] Hao Q., Wang J., Niu J. (2009). Study on phytoestrogenic-like effects of four kinds of Chinese medicine including Radix Rehmanniae Preparata, Radix Paeoniae Alba, Radix Angelicae Sinensis, Rhizoma Chuanxiong. *China Journal of Chinese Materia Medica*.

